# IUC24420-77 Prognostic impact of ABCA1/ABCG1 expression, lipid parameters, and nephrectomy in patients with renal cell carcinoma receiving immune checkpoint inhibitors

**DOI:** 10.1093/oncolo/oyaf248.016

**Published:** 2025-08-29

**Authors:** G C Giudice, M Maffezzoli, L Gnetti, P Tuttobene, F Pecci, G Pinterpe, S Travaglini, R Marchitelli, F Perrone, S Buti

**Affiliations:** Medical Oncology Unit, Comprehensive Cancer Center of Reggio Emilia, Reggio Emilia, Italy; Medical Oncology Unit, University Hospital of Parma, Parma, Italy; Pathology Unit, University Hospital of Parma, Parma, Italy; Medical Oncology Unit, University Hospital of Parma, Parma, Italy; Department of Medicine and Surgery, University of Parma, Parma, Italy; Clinica Oncologica e Centro Regionale di Genetica Oncologica, AOU delle Marche, Ancona, Italy; Clinica Oncologica e Centro Regionale di Genetica Oncologica, AOU delle Marche, Ancona, Italy; Clinica Oncologica e Centro Regionale di Genetica Oncologica, AOU delle Marche, Ancona, Italy; Medical Oncology Unit, University Hospital of Parma, Parma, Italy; Medical Oncology Unit, University Hospital of Parma, Parma, Italy

## Abstract

**Background:**

Despite advances with immune checkpoint inhibitors (ICIs) in metastatic renal cell carcinoma (mRCC), many patients do not achieve durable responses. The CHOMET study aimed to evaluate prognostic factors in patients receiving first-line ICIs, focusing on tumor expression of cholesterol transporters (ABCA1/ABCG1), lipid and nutritional status, and the role of nephrectomy.

**Methods:**

CHOMET was a single-center retrospective study of patients with mRCC treated with first-line ICI-based combinations. Immunohistochemistry assessed ABCA1 and ABCG1 expression. Baseline data included serum lipids, albumin, CONUT score, neutrophil-to-lymphocyte ratio (NLR), and nephrectomy status. Progression-free survival (PFS) and overall survival (OS) were analyzed using Kaplan–Meier and Cox regression. Associations were tested with linear regression and Mann–Whitney tests.

**Results:**

Among 86 patients (median follow-up: 30 months), median PFS was 25.6 months and OS was not reached. High ABCA1/ABCG1 expression correlated with shorter PFS and OS. Better outcomes were observed in patients with lower CONUT score, cholesterol ≥200 mg/dL, and triglycerides ≥150 mg/dL. Nephrectomy, performed in 59% of patients, was associated with significantly better survival and favorable baseline lipid and nutritional profiles. A combined nephrectomy-cholesterol score (0-2 points) stratified prognosis: higher scores predicted longer survival (Table 1). In multivariable analysis, both nephrectomy and high cholesterol were independently associated with reduced risk of progression and death.

**Conclusions:**

In mRCC, high tumor expression of ABCA1/ABCG1 predicted poorer outcomes, while favorable metabolic and nutritional profiles and nephrectomy predicted improved survival. The nephrectomy-cholesterol score may serve as a prognostic tool and highlight the relevance of host metabolic status in ICI response.

**Table 1.** Oncological outcomes according to ABCA1/G1 staining intensity, cholesterol and triglycerides levels, CONUT, and nephrectomy-cholesterol scores.

	**mPFS months (95%CI)**	**mOS months (95%CI)**
** ABCA1 **		
** 1+ **	30 (26-NR)	NR
** 3+ **	5 (3-NR)	13 (8-NR)
***P*** **value**	*.261*	***.014***
** ABCG1 **		
** 1+ **	63 (NR-NR)	NR
** 3+ **	9 (3-NR)	12 (7-NR)
***P value***	***.005***	***.008***
** CONUT score **		
** <3 **	30 (26-NR)	NR
** ≥3 **	10 (3-34)	23 (13-NR)
***P*** **value**	***.005***	***.003***
** Nephrectomy-Cholesterol score **		
** 0 **	10 (6-NR)	23 (12-NR)
** 1 **	27 (24-NR)	NR
** 2 **	33 (26-NR)	NR
***P*** **value**	***.003.029***	***.003.028***

Abbreviations; mOS, median overall survival; mPFS, median progression-free survival; NR, not reached.

